# Metaproteomics: Much More than Measuring Gene Expression in Microbial Communities

**DOI:** 10.1128/mSystems.00115-19

**Published:** 2019-05-21

**Authors:** Manuel Kleiner

**Affiliations:** a Department of Plant and Microbial Biology, North Carolina State University, Raleigh, North Carolina, USA

**Keywords:** LC-MS, metagenomics, metaproteomics, metatranscriptomics, microbiome, microbiota, proteomics, symbiosis

## Abstract

Metaproteomics is the large-scale identification and quantification of proteins from microbial communities and thus provides direct insight into the phenotypes of microorganisms on the molecular level. Initially, metaproteomics was mainly used to assess the “expressed” metabolism and physiology of microbial community members. However, recently developed metaproteomic tools allow quantification of per-species biomass to determine community structure, *in situ* carbon sources of community members, and the uptake of labeled substrates by community members. In this perspective, I provide a brief overview of the questions that we can currently address, as well as new metaproteomics-based approaches that we and others are developing to address even more questions in the study of microbial communities and plant and animal microbiota. I also highlight some areas and technologies where I anticipate developments and potentially major breakthroughs in the next 5 years and beyond.

## Abstract (precis)

Metaproteomics is the large-scale identification and quantification of proteins from microbial communities and thus provides direct insight into the phenotypes of microorganisms on the molecular level. Initially, metaproteomics was mainly used to assess the “expressed” metabolism and physiology of microbial community members.

## PERSPECTIVE

I recently taught a class entitled “Microbial Symbiosis and Microbiomes.” After discussing the myriad of approaches for studying microbial communities and host-microbe interactions, I provided an overview of a personal favorite—metaproteomics. The students seemed enthusiastic about the unique capabilities of metaproteomics, but one student raised a valid question: “If metaproteomics is such a great approach, why aren’t more scientists using it?”

**FIG 1:**
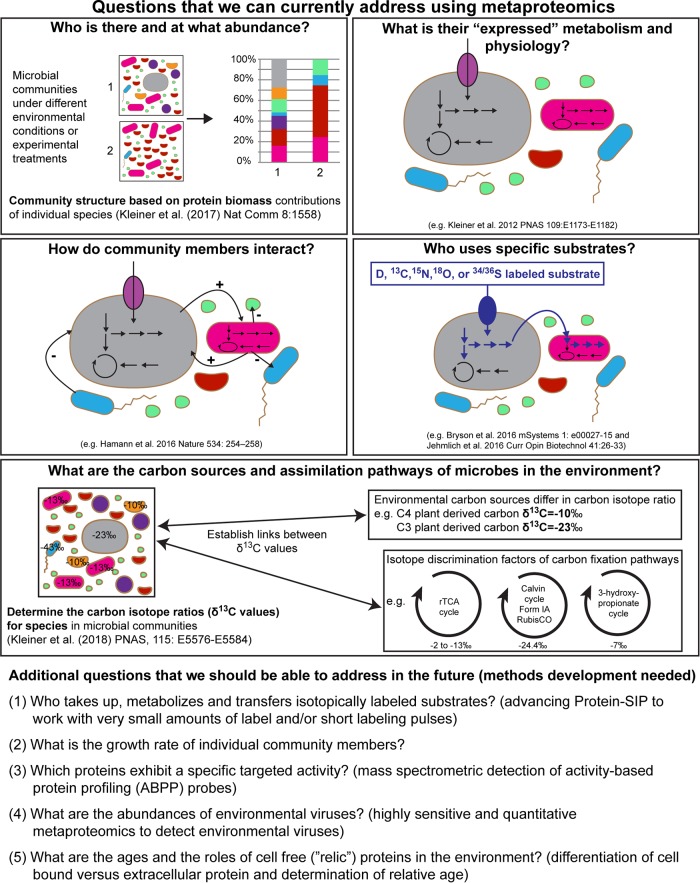
Questions that can or will be addressed with metaproteomics.

Since its inception in 2004 as “the large-scale characterization of the entire protein complement of environmental microbiota at a given point in time” (1), only ∼500 publications in PubMed include “metaproteom*” (compared to >10,000 for metagenomics). Since 2012, the number of publications that mention metaproteomics has grown exponentially, partly due to quantum leaps in enabling technologies that have made metaproteomics more feasible and affordable. Technological advances have mostly occurred in the realm of liquid chromatography (LC) enabling separation of highly complex peptide mixtures, high-resolution mass spectrometry (MS) enabling acquisition of large numbers of accurate mass spectra, and computational tools for data processing and analyses. While in 2004 it was standard practice to separate proteins on a 2D gel and then manually pick individual protein spots for mass spectrometric analyses (a laborious and low-throughput method), we now routinely employ LC-MS/MS approaches to identify and quantify tens of thousands of peptides and >10,000 proteins per sample. The underlying advances in liquid chromatography and high-resolution mass spectrometry have also driven the development of new metaproteomic approaches that enable researchers to address a whole array of questions (Fig. 1).

## QUESTIONS THAT CAN BE ADDRESSED ONLY THROUGH METAPROTEOMICS

The aim of this perspective is not to convince the reader that metaproteomics is the best or only approach to study what transpires in microbial communities. The aim is to highlight the strengths of metaproteomics, which integrates with and often relies on other approaches. For example, building a reference database through metagenomic sequencing is often a prerequisite for metaproteomics (2).

In its basic form, metaproteomics allows us to study the presence and abundances of proteins in any microbial community. With a well-curated protein sequence database in hand, we can assign these proteins to individual species or higher taxa and understand the functional roles and interactions of individual members in the community. Since proteins convey structure and activities to cells, knowing their abundances provides a picture of cellular phenotypes on the molecular level. Pure culture studies from a diversity of organisms have shown that the abundance of a protein indicates its relevance and activity under a given condition. Applying this at the community level can lead to critical insights and significant progress. For example, in a marine worm that relies on its five bacterial symbionts for all nutrition, we did not know which environmental energy sources the symbionts were using for carbon fixation. A metaproteomic study revealed that some of the symbionts abundantly expressed carbon monoxide dehydrogenases, which suggested they were using carbon monoxide as an energy source (3). We were subsequently able to confirm this hypothesis through physiological incubation experiments and nanoscale secondary-ion mass spectrometry (NanoSIMS) measurements (4).

Differential metaproteomics can be used to identify changes in the expression of individual genes, for example, after actively manipulating a microbial community by changing substrates or if environmental conditions naturally fluctuate. We have used this approach to show that hydrogen transfer is the basis of the symbiosis between a *Breviatea* protist and its *Arcobacter* symbiont by comparing metaproteomes from both partners to those of only one partner (5).

The metaproteomic data generated to examine function can also be used to analyze community structure. Protein constitutes the largest amount of cellular material (in most organisms), and thus, total per-species protein can be quantified to assess biomass contributions of individual community members. We recently developed and validated such an approach, which uses standard metaproteomic data to analyze community structure on the basis of biomass instead of gene/genome copy counts (e.g., 16S rRNA amplicon or metagenome sequencing) (6).

If the discovery of uncharacterized proteins with specific enzyme functions is of interest, activity-based probes (ABP) can be used to enrich proteins with specific functions from environmental samples prior to metaproteomic analysis. This approach has, for example, led to the discovery of overrepresented microbial proteases in the intestinal microbiota of mice with inflammatory bowel disease (7).

The high-resolution mass spectrometry data produced by most metaproteomics approaches can also be used to analyze the isotope content of individual proteins and species. Based on this, we have developed a highly sensitive protein stable isotope fingerprinting (direct Protein-SIF) method to analyze natural ratios of carbon stable isotopes, which allows one to determine carbon sources of individual microorganisms in microbial communities, as well as carbon assimilation pathways used by autotrophic organisms (8). Others have used similar approaches to follow the incorporation of isotopically labeled substrates by individual community members using a metaproteomic approach called protein-based stable isotope probing (Protein-SIP) (9, 10). These approaches have much higher resolution and throughput than other strategies that enable isotopic analyses of environmental microorganisms such as NanoSIMS and DNA-SIP.

## QUESTIONS THAT WE PLAN TO ADDRESS IN THE FUTURE

Despite metaproteomics having been around for >15 years, it is still in its infancy and there is great potential for its continued development to address otherwise intractable questions.

In my laboratory, we develop and improve metaproteomic methods to study animal- and plant-associated microbiota with a focus on understanding critical interactions between all partners, including the host. Such studies are challenging for multiple reasons, including the presence of fecal or soil-derived substances that interfere with sample preparation and the high abundances of host-associated proteins that potentially swamp out the microbial signal. In the past we have addressed these challenges by applying prefractionation methods such as density gradient centrifugations and filtrations (3, 11). However, sample preparation and analytical methods have improved so that in most cases samples need not be prefractionated and we can analyze microbial and host gene expression within the same sample. Future improvements in metaproteomic coverage are expected to come with further technological advances (see below).

Together with our collaborators, we are developing a variety of approaches that rely on metaproteomics to address novel questions. These approaches include (i) following the flow of carbon, nitrogen, oxygen, and hydrogen in microbial communities by using highly sensitive Protein-SIP to measure their incorporation into proteins. The high sensitivity will allow working with substrates where only a small fraction is labeled with heavier isotopes rather than the larger fractions that were necessary with earlier versions of this method. The approaches also include (ii) estimating growth rates of community members based on protein turnover rates, (iii) identifying uncharacterized proteins with specific enzymatic functions (as determined by binding of highly specific probes) using activity-based protein profiling with mass spectrometric detection of the probes in metaproteomics, (iv) detecting environmental viruses with higher sensitivity, and (v) differentiating cell-bound from extracellular protein (secreted and relic proteins) in environmental samples. The last approach will be critical to better understand the role of protein in a diversity of environments, as it can be expected that, similar to relic DNA (12), relic/extracellular protein may influence our measurements of environmental gene expression. In contrast to DNA, however, understanding extracellular protein is critical not only from a perspective of impacting our measurements. If it retains activity, this protein could have major impacts on biogeochemical cycling (13).

## FUTURE AREAS FOR DEVELOPMENT AND POTENTIAL BREAKTHROUGHS

In my opinion, there are several developments that will drive metaproteomics advances in the next 5 years and beyond. These include the appearance of new technologies, training additional personnel on metaproteomics approaches, validating and standardizing approaches, developing custom computational tools, and establishing dedicated metaproteomics meetings.

One of the technological factors that limits metaproteomics in terms of throughput is the number of mass spectra that can be acquired by an instrument per unit of time. Currently, most instruments measure, at most, 40,000 spectra per hour, which necessitates several hours of instrument time for an in-depth community analysis. New generations of mass spectrometers with increased measurement frequency will lead to increased metaproteomic throughput. For example, a recent study demonstrated the measurement of 600,000 spectra within 1 h on an instrument that combines ion mobility and time of flight mass spectrometry (14).

Technological innovation could also come from a completely different direction. New technologies are being developed to directly sequence single protein molecules by an approach similar to the DNA sequencing technology developed by Oxford Nanopore (15). Direct sequencing of intact protein molecules could be a game changer, particularly for the difficult task of distinguishing proteins with similar sequences. Protein sequencing would eliminate our reliance on a so-called bottom-up (meta-proteomics) approach in which the identification of protein fragments (peptides) is used to ultimately infer the presence of proteins. Currently, there is no adequate mass spectrometric approach to analyze intact proteins (top-down approach) from complex mixtures, and this will likely not change in the foreseeable future.

The number of research labs developing or using metaproteomics has increased steadily over the last few years, and this has led to an increased number of students and postdocs acquiring the skills necessary to generate and analyze metaproteomic data sets. The growth in the metaproteomic community has also led to an increased demand for bioinformatics tools specifically designed to work with these data. While most data can be analyzed with standard proteomic tools, the emergence of tools specifically developed for metaproteomic data, such as MetaProteomAnalyzer (16), Unipept (17), and Calis-p (8), is promising, as is the integration of metaproteomic data with multi-omic data sets (e.g., references 18 and 19). This trend is expected to continue, and more tools will become available.

The growth of the community has also led us to recognize the need for better validation and standardization of metaproteomic approaches. Currently, there are as many approaches for metaproteomics as there are labs doing metaproteomics, and it is often unclear in publications which approaches work better for particular questions or sample types. Although a few recent articles used fully controlled samples to validate and optimize metaproteomic approaches (6, 20, 21), much more effort is needed in this realm to make metaproteomics more broadly applicable and comparable across laboratories. Fortunately, the increase in the number of labs that do metaproteomics also means that the critical mass was reached a few years ago to establish a dedicated conference, which facilitates the exchange of ideas and discussion on how to standardize and compare approaches. The most recent meeting was the 3rd International Metaproteome Symposium hosted in December 2018 in Leipzig by a team from the Centre for Environmental Research (UFZ). The next meeting will be in summer 2020 hosted by the group of Paul Wilmes in Luxembourg. I look forward to meeting many new metaproteomics enthusiasts there!
